# LIN28B Promotes Cancer Cell Dissemination and Angiogenesis

**DOI:** 10.1002/adbi.202400730

**Published:** 2025-07-18

**Authors:** Diana Corallo, Sara Menegazzo, Marcella Pantile, Silvia Bresolin, Carlo Zanon, Alessandro Davini, Massimiliano Mazzone, Alessandra Biffi, Sanja Aveic

**Affiliations:** ^1^ Laboratory of Target Discovery and Biology of Neuroblastoma Pediatric Hematology Oncology and Hematopoietic Cell&Gene Therapy Research Area Institute of Pediatric Research Fondazione Città della Speranza Padua 35127 Italy; ^2^ Division of Pediatric Hematology Oncology, and Stem Cell Transplant Department of Woman's and Child's Health University of Padua Padua 35128 Italy; ^3^ Institute of Pediatric Research Fondazione Città della Speranza Padua 35127 Italy; ^4^ Laboratory of Tumor Inflammation and Angiogenesis VIB KU Leuven Center for Cancer Biology Leuven 3000 Belgium; ^5^ Pediatric Hematology Oncology and Hematopoietic Cell&Gene Therapy Research Area Institute of Pediatric Research Fondazione Città della Speranza Padua 35127 Italy; ^6^ Department of Dental Materials and Biomaterials Research RWTH Aachen University Hospital 52074 Aachen Germany

**Keywords:** angiogenesis, LIN28B, migration, neuroblastoma, pre‐metastatic niche

## Abstract

Neuroblastoma represents a major challenge in pediatric oncology with over 50% of cases involving metastasis. High‐risk patients face an unfavorable prognosis, with survival rates below 40%. LIN28B plays a pivotal role in neuroblastoma development, being overexpressed in a subset of high‐risk patients with widespread metastases. Here, the effect of induced LIN28B (iLIN28B) expression on neuroblastoma cells is investigated with a focus on key aspects of the metastatic cascade including anchorage, migration, invasion, and angiogenesis. iLIN28B cells show substrate‐selective adherence, coating‐dependent migration, and the context‐guided ability to degrade the extracellular matrix. In response to tumor cell‐derived IGF2, endothelial cells show enhanced motility and proliferation, while inhibition of IGF2 activity impairs LIN28B‐induced angiogenesis in vitro and in vivo. These findings underscore the hub role of LIN28B in favoring pre‐metastatic processes in neuroblastoma. The intricate interplay between LIN28B, endothelial cells, and the extracellular matrix contributes to the development of the aggressive neuroblastoma phenotypes.

## Graphical abstract

Children diagnosed with high‐risk neuroblastoma have a 5‐year event‐free survival rate of less than 50% and poor outcomes after recurrence. Deregulation of the *LIN28B* oncogene can be addressed in these patients. Upregulation of *LIN28B* is shown to support the metastatic cascade. By regulating the IGF2‐IGF1R axis, LIN28B triggers angiogenesis and facilitates the spread of cancer cells.

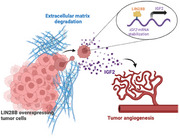

## 1 Introduction

Neuroblastoma is a pediatric tumor of the developing peripheral sympathetic nervous system, originating from the neural crest (NC) cells.^[^ ^1^ ^]^ It is the most common extracranial cancer in children under one year of age and accounts for almost one‐third of all infant cancers.^[^ ^2^ ^]^ Neuroblastoma is a complex disease that can result in diverse clinical outcomes.^[^ ^3^ ^]^ At diagnosis, about half of the patients are clinically stratified within the high‐risk (HR) group that is characterized by a metastatic, and aggressive, disease with an overall survival rate of less than 50% in the five‐year follow‐up period.^[^ ^1^, ^2^ ^]^ Moreover, neuroblastoma is a low mutation burden disease, and there is not a single genetic marker that accurately captures the complex clinical situations described earlier.^[^ ^3^ ^]^ To date, understanding the biology that sustains metastatic behavior and aggressive neuroblastoma cell phenotypes remains a challenge for developing targeted therapies and improving outcomes for HR cases.

The RNA‐binding protein LIN28B coordinates developmental timing and stem cell identity by suppressing the let‐7 family of microRNAs.^[^ ^4^ ^]^ Small nucleotide polymorphisms (SNPs) of *LIN28B* gene have been identified in neuroblastoma.^[^ ^3^ ^]^ In addition, elevated LIN28B expression is associated with aggressive forms of this neoplasm and correlates with a poor prognosis.^[^ ^3^, ^5^ ^]^ Due to its multiple functions, ectopic expression of LIN28B is sufficient to trigger cell transformation and subsequently maintain invasiveness required for metastatic disease formation.^[^ ^6^ ^]^ We have previously reported the importance of LIN28B in regulating the migration and differentiation of the NC of the trunk towards the sympathoadrenergic lineage in the two vertebrate models, *Xenopus leavis* and *Danio rerio*. This occurred due to boosted epithelial‐to‐mesenchymal transition (EMT) in neuroblastoma cells, leading to tumor progression. Moreover, the Src/PI3K/AKT regulatory axis was triggered by the up‐regulation of α5 and α6 integrins sustaining the metastatic phenotypes in LIN28B overexpressing cells.^[^ ^6^ ^]^

Angiogenesis plays a decisive role in tumor progression. The dynamics of angiogenic balance in neuroblastoma are intricated and involve the coordinated activity of a spectrum of stimulators and inhibitors.^[^ ^7^ ^]^ Elevated tumor vascularity is linked to a disseminated disease, *MYCN* amplification, unfavorable tumor histology, and poor patient outcome.^[^ ^8^ ^]^ The insulin‐like growth factor 2 (IGF2) and its two receptors, Insulin‐like Growth Factor 1 Receptor (IGF1R) and insulin receptor (IR), have been shown to play a crucial role in promoting angiogenesis and the progression of cancer.^[^ ^9^ ^]^

IGFs, particularly IGF1 and IGF2, are critical factors for the proliferation, survival, and differentiation of neural stem cells and glial cells in the nervous system, mostly through the activation of PI3K‐AKT and MAPK signaling pathways.^[^ ^10^ ^]^ In neuroblastoma, increased IGF2 level has been correlated with poor prognosis and advanced tumor stage.^[^ ^11^ ^]^ Also, IGF1R signaling has been shown to play an important role in promoting neuroblastoma genesis and in inhibiting apoptosis. IGF1R is expressed in 86% of primary neuroblastoma tumors,^[^ ^12^ ^]^ forming an autocrine loop with IGF2 that serves as its activating ligand.^[^ ^13^ ^]^ In addition, IGF1 supplied by the tumor stroma supports proliferation, motility^[^ ^14^ ^]^ and survival^[^ ^15^ ^]^ of neuroblastoma cells. The binding of IGF1 to IGF1R has been implicated in regulating neuroblastoma metastasis to bone,^[^ ^16^ ^]^ highlighting the multifaceted role of the IGF1/IGF2‐IGF1R axis in disease progression and dissemination.

In this study, we explored the role of the oncogene LIN28B in the development of a pre‐metastatic niche in neuroblastoma. We investigated the effects of induced LIN28B (iLIN28B) expression on the adhesion affinity and interaction of neuroblastoma cells with specific extracellular matrix (ECM) proteins and examined their interplay with nearby endothelial cells. Furthermore, we assessed the mechanisms by which long‐term iLIN28B expression affects post‐transcriptional regulation and secretion of IGF2 and monitored modulation of endothelial cell network formation in vitro and in vivo.

## 2 Results

### 2.1 LIN28B Modulates Pathways Involved in Cell Locomotion and Interaction with the ECM

**Figure 1:**
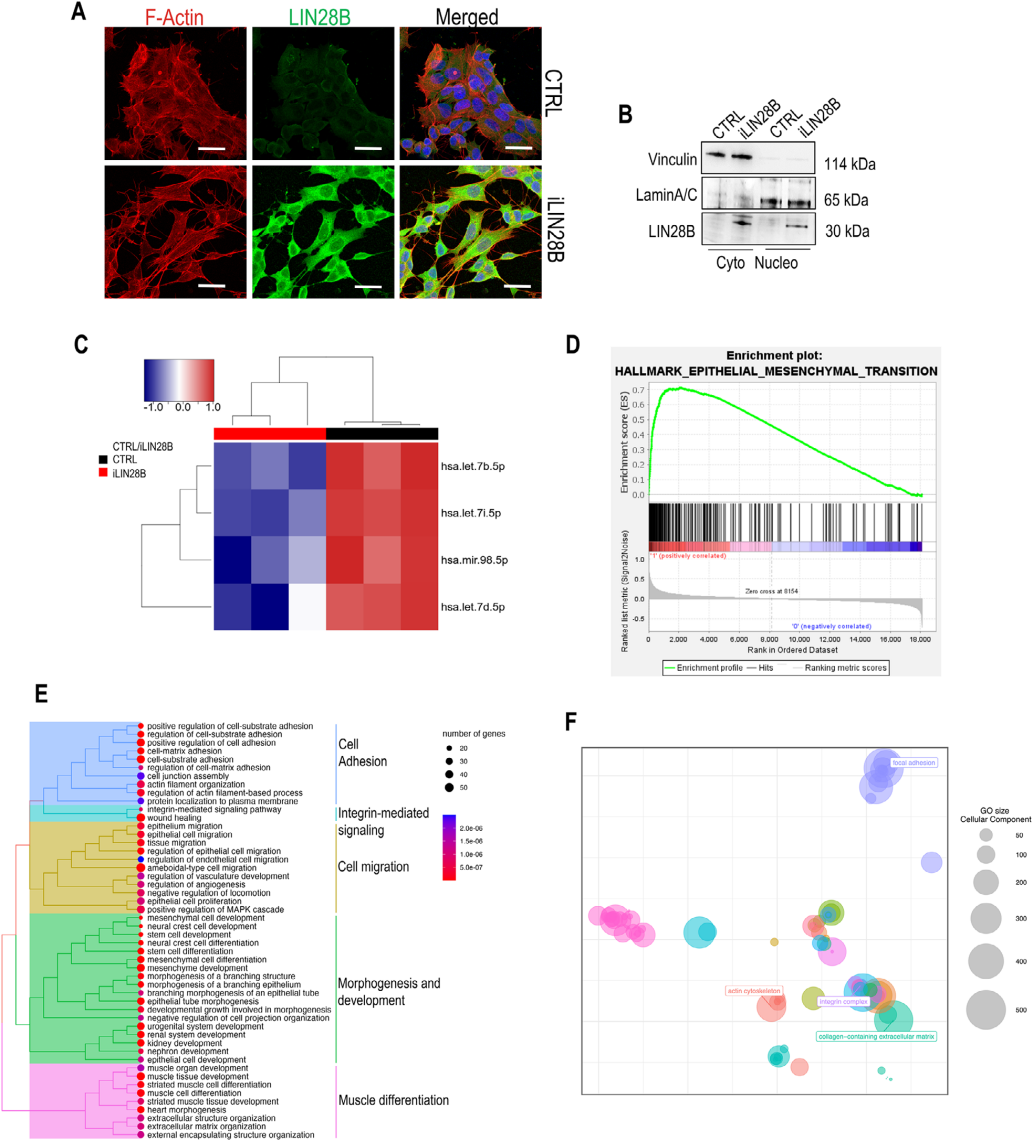
Gene expression analysis in iLIN28B neuroblastoma. A) Immunostaining analysis of LIN28B protein subcellular localization in CTRL and iLIN28B cells. Green—LIN28B; Red—F‐Actin; Blue— DAPI (nuclear staining). Scale bar, 50 µm. B) Immunoblot assay for the verification of LIN28B protein expression and subcellular localization: cytoplasmic (Cyto) and nuclear (Nucleo) fractions were analyzed and Vinculin and LaminA/C served respectively as loading controls. The molecular weight of proteins is reported in KiloDalton (kDa). C) Heat map clustering of significantly changed microRNAs (miRNAs) in iLIN28B and control (CTRL) cells after 7 d of DOX induction. Each column represents an individual cell sample. Red columns are iLIN28B cells (*n* = 3) and black columns are control cells (CTRL, *n* = 3). The overexpressed miRNAs are shown in red and underexpressed miRNAs in blue. D) Gene set enrichment analysis (GSEA) of LIN28B‐dependent mRNAs for gene set involved in the EMT. Each black line represents a single gene in the gene set. Significance set at FDR < 0.05. FDR—false discovery rate. E) Treeplots summarizing over‐representation analysis in GO‐BP gene sets for significantly upregulated genes in iLIN28B cells. F) Scatter plot of the GO cellular component level overrepresentation for iLIN28B upregulated genes after reduction of redundancy among terms. Each dot represents a cluster of redundant GO terms, whereas color represents a super‐cluster of loosely related terms (super‐clusters’ name indicated in color‐font for 4 categories). Distances between points represent the similarity between terms, and axes are the first 2 components of applying a PCoA to the (di)similarity matrix. The size of bubbles represents the number of genes for each term.

The tetracycline‐directed (Tet‐On) gene expression system was used to generate an in vitro inducible *LIN28B* (*iLIN28B*) neuroblastoma cell model having LIN28B protein overexpressed in both subcellular fractions, cytosolic and nuclear (**Figure** **1**A,B). The validity of the proposed Tet‐On system was documented in^[^ ^6^ ^]^ and confirmed here by analyzing the expression of LIN28B target miRNAs, let‐7b‐5p, let‐7i‐5p, let‐7d‐5p, and miR98‐5p miRNAs,^[^ ^5^ ^]^ all of which were significantly decreased (Figure 1C). To investigate key genes affected by long‐term iLIN28B expression, an in‐depth analysis of the transcriptome of neuroblastoma cells was performed. A total of 576 transcripts were found as differentially expressed in iLIN28B compared to CTRL samples. Gene Set Enrichment Analysis (GSEA) corroborated the involvement of genes regulating EMT (Figure 1D), and the involvement of LIN28B in promoting the phenotypic transition of neuroblastoma cells towards a mesenchymal form with increased migratory capacity.^[^ ^6^, ^17^ ^]^ Given that 98% of differentially expressed genes were found overexpressed in iLIN28B cells, a hierarchical clustering of overrepresented enriched terms was calculated. The identified genes were organized into five distinct subtrees, corresponding to interconnected biological processes associated with cell adhesion and migration, integrin‐mediated signaling, and development (Figure 1E). In correspondence, the cellular components determined by shared genes with the highest expression were predominantly associated with the actin cytoskeleton, focal adhesion, integrin complex, and collagen‐containing ECM (Figure 1F).

### 2.2 LIN28B Defines a Selective Cell–Substrate Adherence

**Figure 2:**
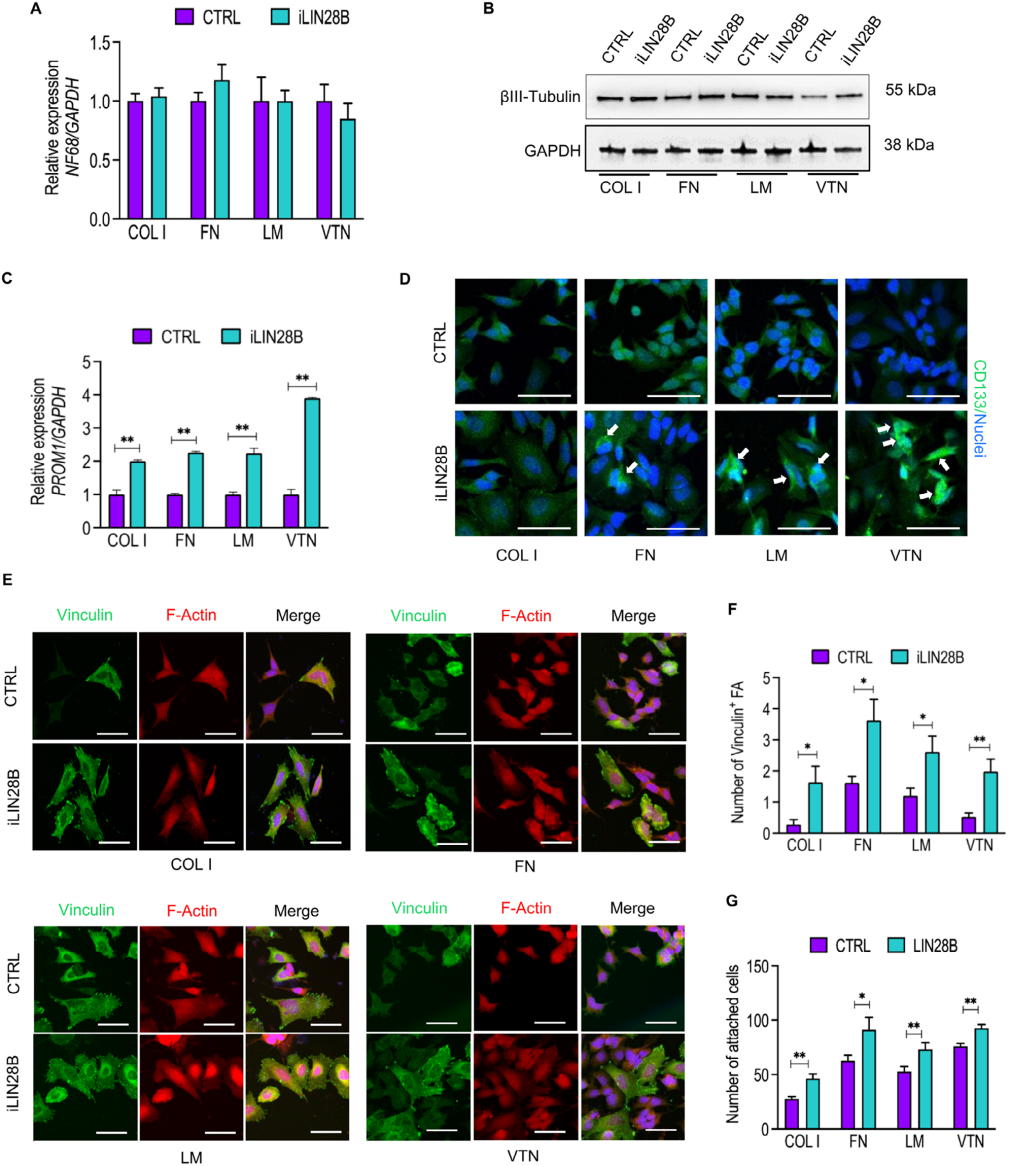
Surface‐coating proteins enhance iLIN28B cell adhesion and stemness. A) Real‐time qPCR analysis for *NF68* mRNA on CTRL and iLIN28B cells grown on the indicated coatings. B) Immunoblot analysis for the differentiation marker βIII‐Tubulin on CTRL and iLIN28B cells grown on the indicated coatings. An antibody against GAPDH represents loading control. The molecular weights are indicated in KiloDaltons (KDa). C) Real‐time qPCR analysis for *PROM1* mRNA on CTRL and iLIN28B grown on the indicated coatings and normalized to *GAPDH* gene level. D) Immunofluorescence staining for CD133 on CTRL and iLIN28B cells grown on the indicated coatings. Nuclei were counterstained with DAPI (blue). Scale bar, 50 µm. E) Representative immunofluorescence analysis of Vinculin (green) and F‐Actin (red) in CTRL and iLIN28B cells grown on the indicated coatings (COL I, FN, LM and VTN). Nuclei were counterstained with DAPI (blue). Scale bar, 50 µm. F) Histogram showing the quantification of Vinculin‐expressing focal adhesions (FA) in CTRL and iLIN28B cells grown on the same substrates as in (E). G) Histogram representing cell adhesion capability of CTRL and iLIN28B cells grown on the indicated substrates. Data are presented as the mean number ± standard error (S.E.M.). *P < 0.05; **P < 0.01 compared to CTRLs (Student's *t*‐test).

iLIN28B neuroblastoma cells induce a significant enrichment of cell‐surface proteins with adhesive functions such as α5 and α6 integrin subunits.^[^ ^6^ ^]^ These data suggest that LIN28B may control a number of genes/proteins expressed by the cells and guide cell specificity for the ECM substrate. To evaluate this hypothesis, we seeded iLIN28B and CTRL cells on different ECM proteins including type I collagen (COL I), laminin (LM), fibronectin (FN), and vitronectin (VTN). First, we evaluated the effects of each coating on cell proliferation rate by evaluating PCNA protein level, and the cell viability by examining the presence of PARP1 protein cleavage. No changes in the level of both markers were detected among iLIN28B and CTRL samples, suggesting that the examined ECM proteins did not affect these cell processes (Figure S1A, Supporting Information). In addition, EdU staining revealed that iLIN28B cells exhibited a proliferation rate comparable to that of CTRL cells under different plating conditions (Figure S1B, Supporting Information). Given that FN, COL I, and LM are known to induce the differentiation of neuroblastoma cells,^[^ ^18^ ^]^ we next investigated whether this phenomenon may be altered in iLIN28B cells. We observed comparable expression levels of the neuronal differentiation markers *NF68* mRNA (**Figure** **2**A) and βIII‐TUBULIN protein between iLIN28B and CTRL cells (Figure 2B). In contrast, the expression of *PROM1, SOX2*, and *NESTIN*, three neuroblastoma stem cell markers,^[^ ^19^, ^20^ ^]^ exhibited a notable increase in all iLIN28B samples compared to their corresponding CTRLs (Figure 2C and Figure S1C, Supporting Information). The observed increase in *PROM1* mRNA was accompanied by enhanced cytoplasmic deposition of the corresponding CD133 protein in iLIN28B cells grown on FN, LM, and VTN, while no such tendency was observed in the cells grown on COL I (Figure 2D).

Treatment of CTRL cells with 13‐cis‐retinoic acid (13‐RA) resulted in a significant downregulation of all assessed stem cell markers, accompanied by a marked upregulation of *NF68*, compared to the DMSO‐treated control (Figure S1D, Supporting Information). However, in iLIN28B‐expressing cells, the pro‐differentiative effect of 13‐RA was significantly attenuated (Figure S1D, Supporting Information). This suggested that LIN28B overexpression impaired the differentiation‐inducing activity of 13‐RA in neuroblastoma cells. Together, these data indicated that LIN28B counteracts the differentiation stimuli provided by FN, LM, and VTN to neuroblastoma cells. However, upon exposure to COL I, this effect is not as pronounced. Moreover, across all examined protein coatings, the number of Vinculin‐positive focal adhesions (FA) was significantly increased in iLIN28B cells (Figure 2E,F). As a result, iLIN28B cells showed enhanced anchorage capability compared to their CTRLs (Figure 2G) sustaining the involvement of LIN28B in the regulation of cell motility process.

### 2.3 The Motility of iLIN28B Cells Is Favored on the Laminin Matrix

**Figure 3:**
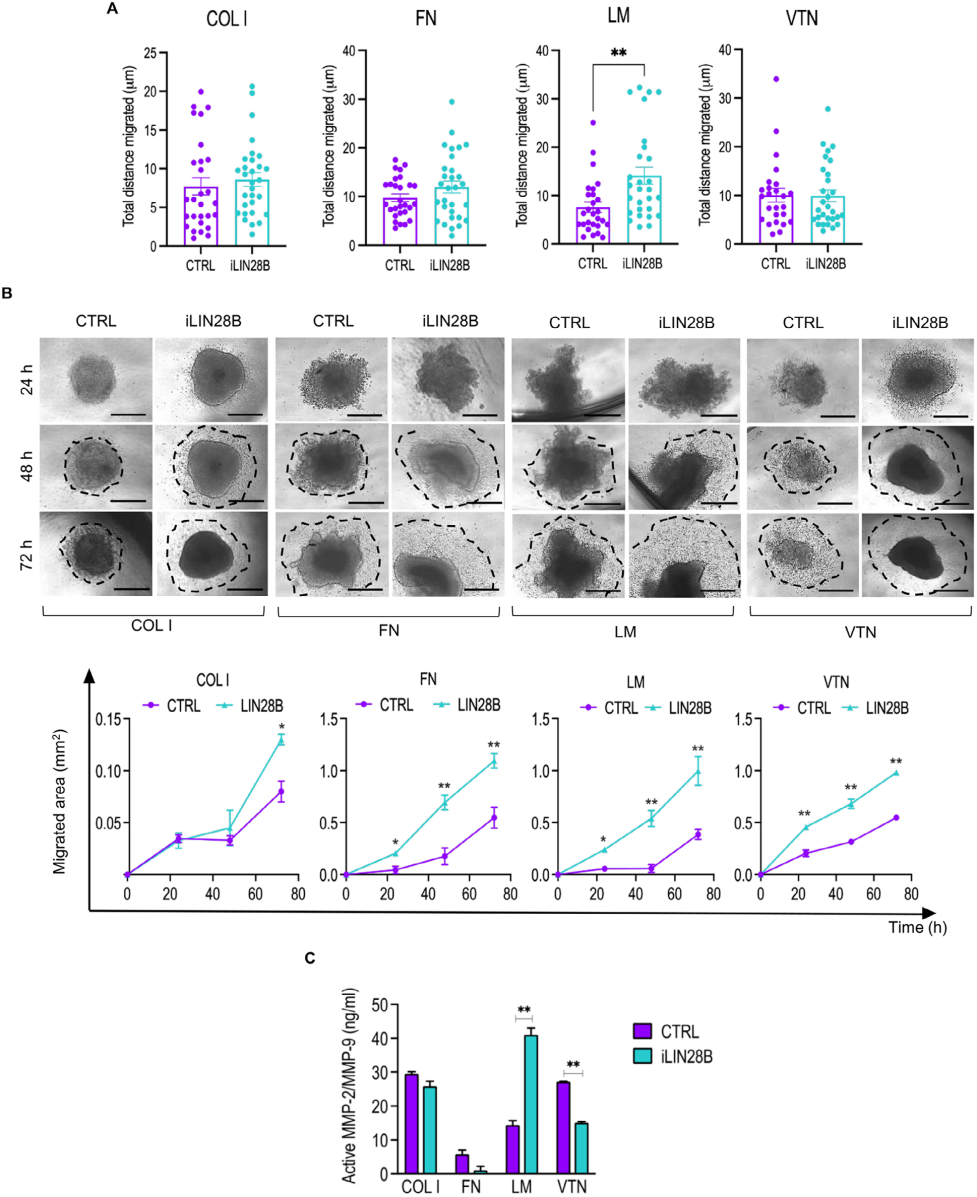
Overexpression of LIN28B influences neuroblastoma cell migration upon specific protein coatings. A) Quantitative analysis of average accumulated distance (µm) of CTRL and iLIN28B cells migrating on the indicated coatings and captured through 8 h time‐lapse microscopy analysis. Each dot represents a migrating cell. B) Representative brightfield images of CTRL and iLIN28B tumor spheroid migration capability after 24, 48, and 72 h growth on the indicated substrates. The leading edge formed by migrating cells is indicated (dotted line). Scale bar, 500 µm. Bottom: quantification of the total migration area covered from the spheroid edge (migrated area, mm^2^). C) Histogram showing the activity of MMP‐2 and MMP‐9 assessed using Innozyme Gelatinase Fluorogenic Activity Assay in CTRL and iLIN28B cells grown on the indicated coatings. Data are presented as the mean number ± standard error (S.E.M.). *P < 0.05; **P < 0.01 compared to CTRLs (Student's *t*‐test).

During the metastatic process, disseminating tumor cells activate specific molecular programs to interact with the surrounding ECM, detach from the primary mass and enter the bloodstream.^[^ ^21^ ^]^ The increased adhesion of iLIN28B cells to the ECM could potentially result in a substrate‐specific modulation of their motility.^[^ ^22^ ^]^ To gain insight into this hypothesis, we compared the migration capability of iLIN28B and CTRL cells on the ECM protein substrates using time‐lapse microscopy. While iLIN28B and CTRL cells migrated a comparable distance on COL I, FN, and VTN coatings, iLIN28B cells approached significantly greater distances on the LM than their CTRL counterpart (**Figure** **3**A). These results demonstrate the affinity of iLIN28B cells for the LM substrate that determines the dynamics of cell migration. To recapitulate a more idealized 3D environment, we explored the functional consequences of the transition from a 2D to a tumor spheroid‐based migration assay.^[^ ^23^, ^24^ ^]^ The growth kinetics of spheroids derived from either iLIN28B or CTRL cells was comparable (Figure S1D, Supporting Information), and each counterpart maintained optimal cell viability within the given time frame (Figure S1E, Supporting Information). However, iLIN28B‐deriving spheroids formed a clearly defined migration edge earlier (within 24 h) than CTRLs on all protein substrates except for COL I (Figure 3B). After 72 h, migration of iLIN28B cells was triggered on the COL I substrate as well, remaining however less attractive in comparison to the other ECM proteins (Figure 3B). This migration front on the LM surface was accompanied by enhanced matrix‐metalloproteinase 2 (MMP‐2) and MMP‐9 enzyme activity in iLIN28B cells (Figure 3C). In contrast, FN and VTN coatings sustained a reduction in MMP‐2/MMP‐9 activity (Figure 3C). Overall, these results suggest a distinct migration program activated by iLIN28B cells in response to different ECM stimuli.

### 2.4 iLIN28B Enhances the Proliferation and Sprouting of Endothelial Cells

**Figure 4:**
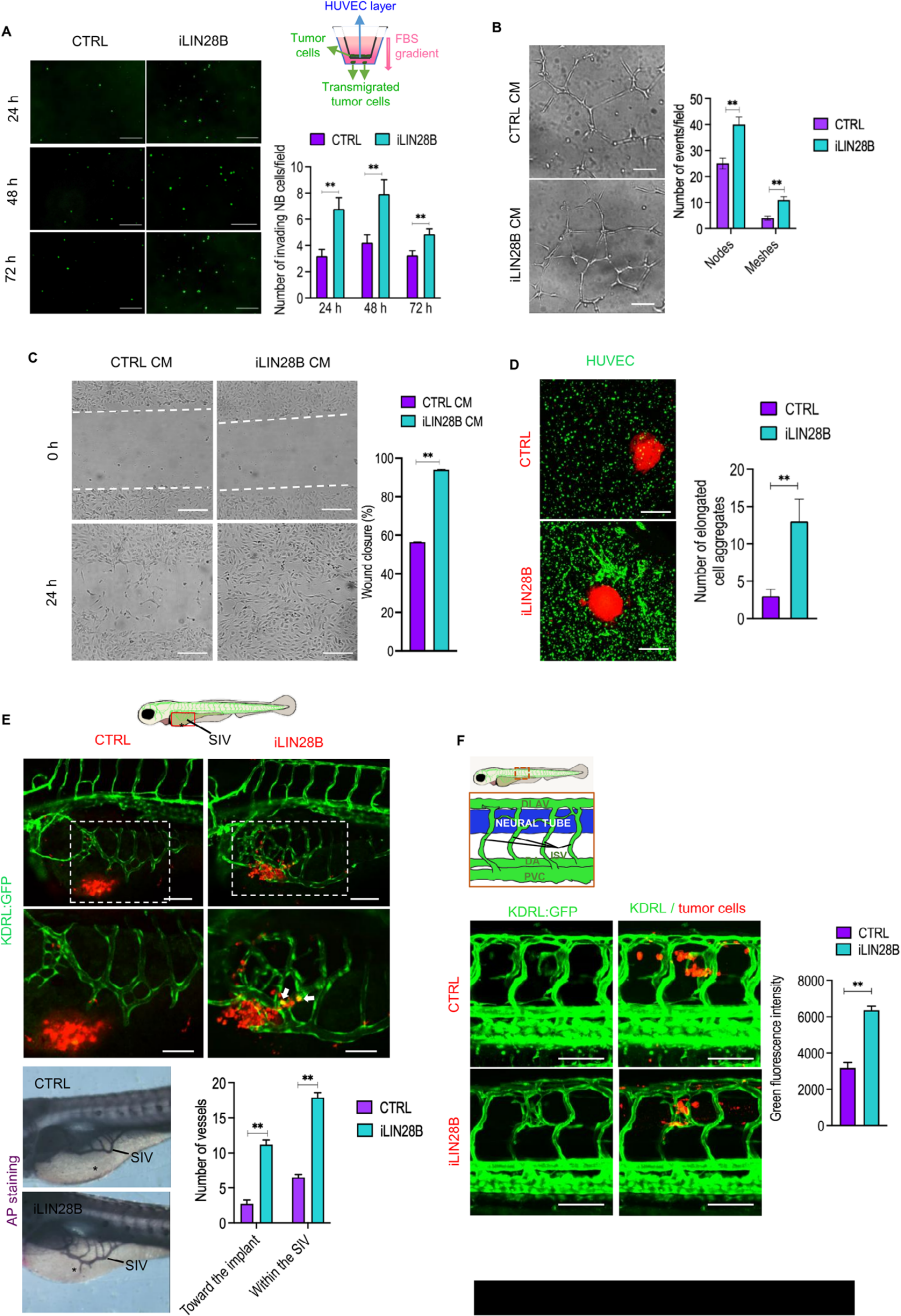
LIN28B promotes angiogenesis and stimulates the migration of endothelial cells in vitro and in vivo. A) Representative images of transmigrated CTRL and iLIN28B cells at the indicated time points (hours, h). Scale bar, 300 µm. Bottom right: quantification of the number of transmigrated cells from at least five random fields. Top right: transendothelial migration assay. CTRL and iLIN28B cells (green) were seeded onto the upper chamber of a transwell with an endothelial monolayer (blue). The FBS gradient stimulated the transendothelial migration of neuroblastoma cells. B) Branching of endothelial cells treated with CTRL and iLIN28B CM. Scale bar, 300 µm. Right: quantification of nodes and meshes. C) Scratch assay with endothelial cells treated with CTRL and iLIN28B conditioned media (CM) for 24 h. Scale bars, 200 µm. The leading fronts are highlighted with dotted lines. Right: quantification of the percentage (%) of wound closure. D) Confocal images of VitroGel hydrogel‐embedded endothelial cells (green) grown in the presence of CTRL and iLIN28B spheroids (red). Scale bars, 500 µm. Right: quantification of elongated endothelial cells. E) Top: zebrafish xenotransplantation model, red box shows the site of analysis including tumor site (asterisk) and the SIV. Middle: confocal Z‐stack reconstructions of Tg(KDRL:GFP) embryos transplanted with CTRL and iLIN28B cells (red). White arrows indicate intravasated iLIN28B single cells. White dotted squares indicate the area considered. Scale bars, 100 µm (upper panels) and 50 µm (lower panels). *n* = 32. Bottom: Alkaline Phosphatase (AP) staining of xenotransplanted embryos. Bottom right: quantification of the number of vessels toward the tumor mass and within the sub‐intestinal vein. *n* = 30. F) Top: schematic representation of xenotransplanted embryo, the red box indicates the site of analysis comprising the neural tube (NT), the dorsal longitudinal anastomotic vessel (DLAV), intersomitic vessels (ISV), dorsal aorta (DA), and posterior cardinal vein (PCV). Bottom: confocal microscopy images of tumor vasculature stimulated by CTRL and iLIN28B cells (red). Scale bar, 100 µm. Bottom right: quantification of green fluorescence intensity deriving from blood vessels. *n =* 30. Data are presented as the mean number ± standard error (S.E.M.). *P < 0.05; **P < 0.01 compared to CTRLs (Student's *t*‐test).

Angiogenesis is a crucial biological process contributing to tumor cell dissemination and metastasis.^[^ ^8^ ^]^ Besides EMT and cytoskeleton reorganization‐related pathways, our in silico analysis revealed an enrichment of pro‐angiogenic genes in iLIN28B cells (Figure S2A, Supporting Information). To further investigate the involvement of ectopic LIN28B expression in this stage of the metastatic cascade, we conducted a transwell‐based transendothelial assay. In such circumstances, iLIN28B cells showed a greater ability to transmigrate through the endothelial monolayer than CTRL cells at all the time points analyzed (**Figure** **4**A). In addition, the sprouting of endothelial cells was boosted in the presence of iLIN28B‐derived conditioned medium (CM), resulting in the generation of more meshes and nodes compared to CTRLs (Figure 4B). This feature was additionally supported by a more pronounced migration of endothelial cells when cultured with iLIN28B‐CM in 2D conditions (Figure 4C). In a 3D context, we monitored the angiogenic effect of neuroblastoma spheroids implanted in a hydrogel containing endothelial cell. We observed an accumulation of endothelial cells in the proximity of iLIN28B spheroids, whereas no such structures were found for the CTRL spheroids (Figure 4D). Similarly, in the in vivo zebrafish‐tumor xenograft angiogenic model,^[^ ^25^ ^]^ a dense network of blood vessels within the sub‐intestinal venous plexus (SIV) reached the peritumoral region within 24 h (Figure 4E). Importantly, a few iLIN28B cells also intravasated the blood vessels (Figure 4E, white arrows). The same phenomenon was observed in another zebrafish/tumor xenograft angiogenic model in which tumor cells were injected into the neural tube of zebrafish embryos (Figure 4F). The enhanced vascularization observed in response to iLIN28B, but not to CTRL cells, corroborated a strong pro‐angiogenic and vasoinvasion effect of LIN28B in vivo.

### 2.5 IGF2 Is the Major Driver of Tumor Angiogenesis in iLIN28B Neuroblastoma Cells

**Figure 5:**
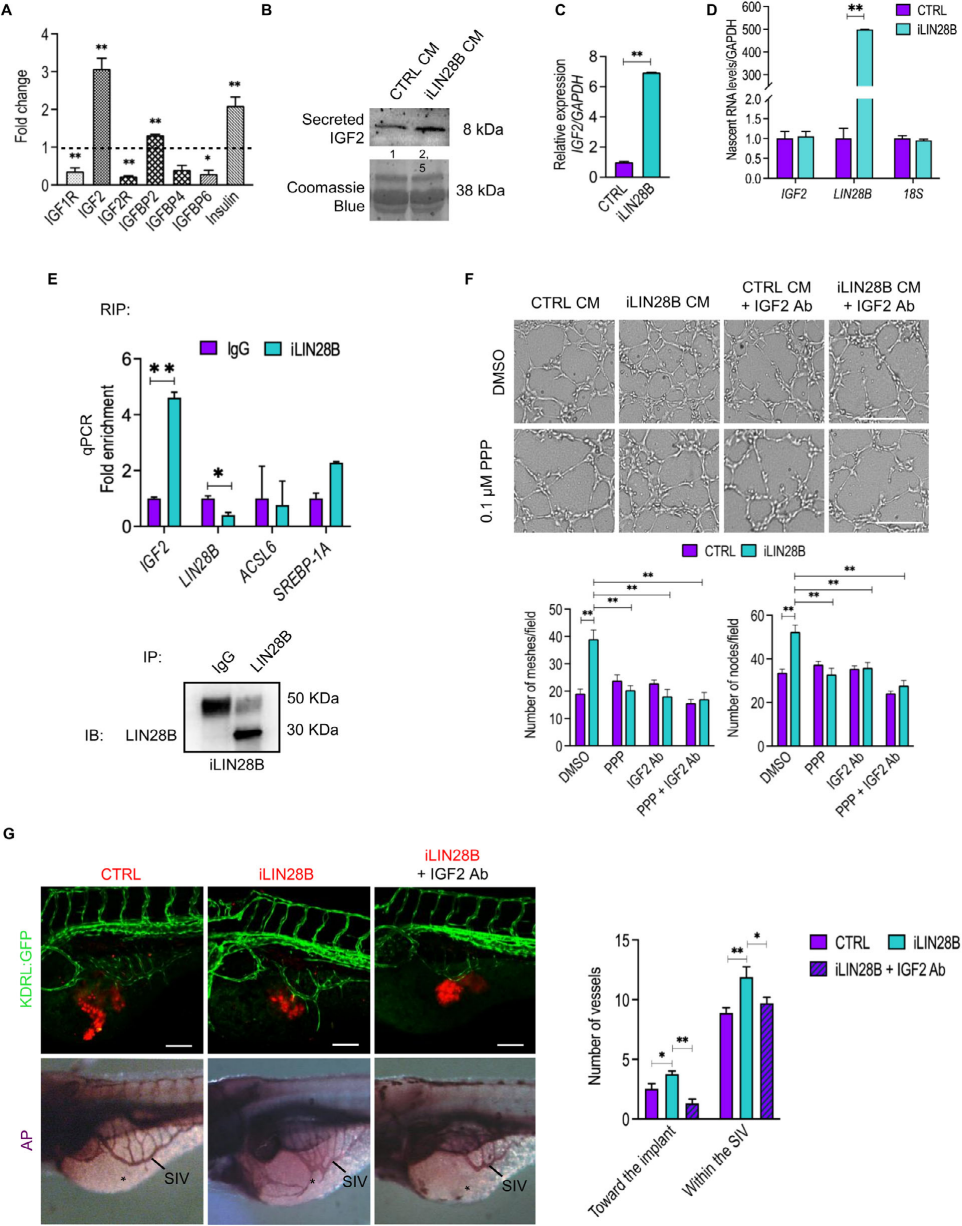
LIN28B promotes angiogenesis through IGF2 secretion. A) The level of IGFs and IGFRs of CTRL and iLIN28B‐derived conditioned media was determined using a Quantibody human IGF signaling array Q1. Fold change values were compared to CTRL (CTRL = 1, dashed line). B) Immunoblot analysis for IGF2 on CTRL and iLIN28B CM. Coomassie‐brilliant‐blue staining was shown as loading control. The molecular weights are indicated in KiloDaltons (KDa). Densitometric analysis of Western blots was performed by ImageJ software and the results were represented as fold change with respect to CTRL CM. C) Real‐time qPCR analysis for total *IGF2* mRNA content in CTRL and iLIN28B cells. The expression of *GAPDH* was used for data normalization. D) Real‐time qPCR analysis of the indicated nascent transcripts in CTRL and iLIN28B cells. The expression of *GAPDH* was used for normalization of each sample. E) The binding of LIN28B with the indicated mRNAs was tested by RIP analysis through real‐time qPCR analysis. Data were then plotted as the fold enrichment of mRNAs in the iLIN28B immunoprecipitated (IP) sample relative to the ones observed in the IgG IP samples. Bottom: the presence of LIN28B in the IP material was confirmed by Immunoblot analysis. F) Top: representative brightfield images of the vessel‐like structures formed by endothelial cells in the presence of iLIN28B and CTRL CM pre‐incubated with IGF2 blocking antibody (IGF2 Ab). Where indicated, endothelial cells were pre‐treated with PPP. Scale bar, 300 µm. Bottom: quantification of meshes and nodes. G) Left: confocal Z‐stack reconstructions of Tg(KDRL:GFP) zebrafish embryos engrafted with CTRL, iLIN28B, and iLIN28B (red) resuspended with IGF2 blocking antibody (IGF2 Ab). For vessel quantification, the embryos were stained for the AP; *n* = 30. Asterisks indicate the site of engraftment. Scale bars, 100 µm. Right: quantification of the mean number of vessels from the SIV towards the tumor mass and within the SIV. Data are presented as the mean number ± standard error (S.E.M.). *P < 0.05; **P < 0.01 compared to CTRLs (Student's *t*‐test).

The angiogenic response of endothelial cells treated with the CM deriving from iLIN28B cells could be achieved by the release of certain soluble factors. To explore this hypothesis, we compared the secretome of iLIN28B and CTRL cells to determine the presence of key angiogenic molecules. We excluded the contribution of the vascular endothelial growth factor (VEGF) in the previously observed angiogenic sprout, since a comparable level of VEGF was detected between iLIN28B and CTRL samples (Figure S2B, Supporting Information). Conversely, the expression of iLIN28B led to a significantly increased secretion of IGF2 and Insulin, both belonging to the IGF signaling pathway (**Figure** **5**A). At gene level, the co‐expression analyses revealed a strong correlation between *LIN28B* and *IGF2* (*R* = 0.99). To functionally validate the contribution of IGF2 protein in our model system, we at first confirmed its increase in iLIN28B‐derived CM (Figure 5B). The increased secretion of IGF2 by iLIN28B cells was also associated by a noteworthy increase of the *IGF2* transcript (Figure 5C). To get insight into the mechanisms of iLIN28B‐dependent IGF2 upregulation, we first investigated whether the increased *IGF2* mRNA levels could be a consequence of its increased transcription.^[^ ^26^ ^]^ While *LIN28B* nascent transcripts were strongly enriched in iLIN28B cells, the *IGF2* nascent mRNAs were comparable in the total pool of each of the analyzed cellular mRNA (Figure 5D). This result indicated the absence of a direct *IGF2* transcriptional regulation by LIN28B oncogene. Given the role of LIN28B as an RNA‐binding protein (RBP),^[^ ^27^ ^]^ we next analyzed whether LIN28B can bind and stabilize *IGF2* mRNA, leading to overexpression of the target protein. The RNA immunoprecipitation (RIP) analysis validated such an interaction between iLIN28B protein and *IGF2* mRNA (Figure 5E) implying the importance of LIN28B oncogene in posttranscriptional regulation of *IGF2* through mRNA stabilization. Next, to impede the activity of IGF2 in CM derived from either iLIN28B or CTRL cells, we employed in vitro and in vivo biological and chemical blocking strategies. In the first case, the addition of an IGF2 neutralizing antibody in iLIN28B CM resulted in a significant depletion of endothelial cell sprouting and migration, while it had little or no effect on endothelial cells exposed to CTRL‐derived CM (Figure 5F and Figure S2C, Supporting Information). Importantly, in none of the analyzed conditions, the viability of endothelial cells has changed (Figure S2D, Supporting Information). Similarly, pre‐treatment of endothelial cells with the picropodophyllin (PPP), a small‐molecule inhibitor of IGF1R, maintained the number of meshes and nodes at the levels found in CTRL cells (Figure 5F). The combined use of the neutralizing IGF2 antibody and PPP did not act synergistically, but either of the events was sufficient to block the iLIN28B‐mediated pro‐angiogenic effect over endothelial cells (Figure 5F). Finally, the use of the IGF2 neutralizing antibody significantly reduced the number of blood vessels (GFP^+^ and AP^+^ signal) and their sprouting from and within the SIV of zebrafish embryos xenotransplanted with iLIN28B cells (Figure 5G). Together, these findings confirm a strong involvement of LIN28B oncogene in favoring the IGF2‐dependent angiogenesis and in facilitating metastatic cell spreading.

## 3 Discussion

The RNA‐binding protein LIN28B is typically expressed during embryogenesis and plays a crucial role in maintaining the identity of stem and progenitor cells primarily by inhibiting the biogenesis of the *let‐7* miRNA family.^[^ ^4^ ^]^ In addition to its physiological function, LIN28B has been implicated in the development of various cancer types, including neuroblastoma.^[^ ^28^ ^]^ Neuroblastoma is a pediatric solid tumor arising from a transient population of stem cells, the NC deriving ones. Neuroblastoma is known for its clinical and biological heterogeneity and its possible presentation as a disseminated disease at diagnosis, leading to a poor prognosis.^[^ ^1^ ^]^ Genomic aberration and overexpression of *LIN28B* oncogene contributes to this phenotype in a subgroup of HR patients with metastatic disease.^[^ ^5^ ^]^ The metastatic process is complex and consists of several steps. The first step allows the metastatic cells to escape from the primary tumor and requires the acquisition of a mesenchymal cell state. Next tumor cell migration relies on the regulation of various biological processes, including actin cytoskeleton dynamics, integrin‐dependent adhesion and proteolytic cleavage of ECM proteins.^[^ ^29^ ^]^ To gain deeper insights into the mechanism of action through which LIN28B sustains tumor cell dissemination, we employed a genetically modified doxycycline‐inducible cell system to achieve ectopically overexpressed LIN28B oncogene in the SH‐SY5Y neuroblastoma cells. This approach allowed us to mimic the conditions of elevated LIN28B mRNA and protein levels verified in a proportion of aggressive neuroblastomas.^[^ ^5^ ^]^ Furthermore, our recent results suggest that sustained overexpression of LIN28B confers a pro‐metastatic phenotype to neuroblastoma cells, enhancing their invasion and migration through activation of EMT and increased expression of the a5 and a6 integrin subunits.^[^ ^6^ ^]^ With respect to ligand specificity, a5 and a6 integrins are respectively classified as arginine‐glycine‐aspartic acid (RGD)‐recognizing and LM‐binding integrins.^[^ ^30^ ^]^ Therefore, it is very likely that the increased migration on FN, VTN, and LM substrates described in this manuscript could be strongly influenced by the overexpression of the a5 and a6 integrin subunits.^[^ ^6^ ^]^ In addition, it is known that during embryonic development, the trunk NC cells express receptors for FN and VTN, both of which contain the RGD motif, as well as for LM.^[^ ^31^ ^]^ It is therefore reasonable to assume that the physiological interaction of NC with the surrounding ECM during the development of the embryonic peripheral nervous system could be awakened by malignant LIN28B neuroblastoma cells during the metastatic process.^[^ ^32^ ^]^

In addition to its permissive role for the migration of NC along the embryonic body, LM represents a key constituent of the basement membrane of the human adrenal cortex (a common primary site of neuroblastoma development),^[^ ^33^, ^34^ ^]^ and of the mature vasculature.^[^ ^35^ ^]^ Here we have shown that, compared to all other matrices tested, the growth of iLIN28B cells on LM coatings significantly increased the activation of the MMP‐2 and MMP‐9 (MMP2/9), which play a central role in ECM degradation and promote metastasis and angiogenesis of neuroblastomas.^[^ ^36^, ^37^ ^]^ These results indicate that LIN28B could potentially support LM‐dependent migration within the adrenal gland during the initial phase of metastasis, promoting MMP2/9‐dependent ECM degradation and leading to the cell detachment from the primary tumor. On the other hand, the increased motility observed on VTN matrix may result from other biological processes not necessarily related or dependent on the MMPs activation. Indeed, as VTN is not deposed in the human adrenal gland,^[^ ^38^ ^]^ the reduced MMPs activity observed upon this ECM protein might be the result of a tissue‐dependent activation of different migratory programs that do not imply matrix degradation. In addition to the role as substrate for cellular migration, the ECM has fundamental roles in modulating cell activities by activating or suppressing specific intracellular signaling pathways.^[^ ^39^ ^]^ In particular, a previous study indicated that SH‐SY5Y neuroblastoma cells differentiate when grown on LM.^[^ ^18^ ^]^ However, our SH‐SY5Y cell model of iLIN28B showed elevated levels of the stem‐related CD133 molecular marker, indicating that LIN28B hijacks the pro‐differentiation stimuli provided by LM. Overall, the LM substrate represents the most challenging matrix on which iLIN28B cells activate part of the metastatic program through increased migration, ECM degradation and angiogenesis.

Tumor angiogenesis, a crucial step of the metastatic cascade, supplies oxygen and nutrients to cancer cells and facilitates the metastatic spread of the disease.^[^ ^40^ ^]^ In neuroblastoma, various mechanisms have been associated with tumor angiogenesis, with the predominant role of secreted growth factors such as VEGF, fibroblast growth factor‐2 (FGF‐2), platelet derived growth factor (PDGF), epidermal growth factor (EGF), and transforming growth factor alpha (TGF‐α).^[^ ^7^ ^]^ The involvement of LIN28B in tumor angiogenesis has been documented in various pathological conditions, including several solid tumors.^[^ ^28^ ^]^ One of the main mechanisms of LIN28B‐mediated tumor angiogenesis is achieved via elevated VEGF production.^[^ ^41^, ^42^ ^]^ Our study revealed that the overexpression of LIN28B in neuroblastoma cells correlates with increased pro‐angiogenic events in 2D and 3D in vitro neuroblastoma cell systems and in vivo in two independent zebrafish xenotransplantation models. In particular, we were able to demonstrate that iLIN28B neuroblastoma cells can stimulate the migration of endothelial cells and the formation of mesh structures through the release of IGF2. This effect is promoted by LIN28B, which stabilizes and maintains IGF2 protein synthesis through direct or indirect binding to *IGF2* mRNA. Here, we describe the LIN28B/IGF2 signaling pathway as a novel mechanism that supports tumor angiogenesis in LIN28B‐dependent neuroblastoma. The effect of IGF2 on endothelial cell migration and angiogenesis is rescued by the addition of an IGF2‐neutralizing antibody, supporting further a functional link between IGF2 signaling and the ectopic LIN28B expression. Furthermore, we found that inhibition of IGF1R on endothelial cells with the small molecule PPP had a similar effect on vessel formation, confirming that inhibition of IGF2‐IGF1R axis is sufficient to prevent the pro‐angiogenic, LIN28B‐dependent angiogenesis. This aspect could have important implications for treatment strategies to overcome pathological angiogenesis induced by the LIN28B oncogene. This could be crucial for neuroblastoma, as the combination of cancer therapeutics and IGF axis inhibitors has been proven to be beneficial in diverse preclinical models.^[^ ^43^ ^]^ Importantly, observed effects were *MYCN* independent since this oncogene did not change in our inducible‐LIN28B cellular model.^[^ ^44^, ^45^ ^]^

Our in vivo studies also sustained the role of LIN28B overexpression in the regulation of neuroblastoma metastasis as confirmed in two different zebrafish/neuroblastoma angiogenesis models.^[^ ^25^ ^]^ The zebrafish model allows the in vivo observation of the multistep metastatic cascade of events, including angiogenesis, tumor‐endothelium cell interaction, and intravasation of cancer cells.^[^ ^25^ ^]^ In our study, the injection of iLIN28B cells in the zebrafish embryo resulted in a robust angiogenic response, with a rapid recruitment of endogenous blood vessels in close proximity to the tumor mass. Moreover, this model allowed the in vivo visualization of single iLIN28B cells intravasating the tumor‐associated blood vessels. This cellular behavior is consistent with the in vitro ability of iLIN28B cells to cross the endothelial barrier and is of central importance in metastases, as migrating cells must increase their transendothelial permeability to successfully cross the endothelial barrier, enter the bloodstream and reach distant body regions. Our study indicates that the overexpression of LIN28B in neuroblastoma facilitates cell migration across the endothelial barrier and augment their angiogenic capacity. These findings align with previous research linking increased vascular density to aggressive neuroblastoma and poor outcomes.^[^ ^46^ ^]^

## 4 Conclusions

In conclusion, our study has provided insights into the role of iLIN28B in sustaining the aggressive phenotypes of neuroblastoma and the process of tumor angiogenesis. Additionally, we demonstrated a link between LIN28B and IGF2 that shed light on their reciprocal role for the formation of a pre‐metastatic niche. Overall, our findings delineate the multiple roles of LIN28B in neuroblastoma metastatic progression, including its involvement in promoting matrix‐selective tumor cell migration and angiogenesis in vitro and in vivo. Understanding these mechanisms may provide potential therapeutic targets for the treatment of the LIN28B‐dependent aggressive forms of this disease, avoiding the problems associated with direct targeting of this RNA‐binding protein, which remains a major challenge.

## 5 Experimental Section

### Cell Culture and Reagents

SH‐SY5Y neuroblastoma cell line bearing doxycycline (DOX)‐inducible LIN28B (iLIN28B) construct and its corresponding control (CTRL) without LIN28B were obtained by a lentiviral infection following the protocol described previously.^[^ ^6^ ^]^ The endothelial cell line was a kind gift from Dr. Elena Porcù (Woman and Child Health Department, Padua University, Fondazione Citta’ della Speranza Istituto di Ricerca Pediatrica, Italy). Endothelial cells were maintained in Medium 200 (Gibco, Thermo Fisher Scientific, Waltham, MA, USA), supplemented with LSGS (Gibco, Thermo Fisher Scientific, Waltham, MA, USA). The CTRL and iLIN28B cells were cultured in RPMI 1640 medium (Gibco, Thermo Fisher Scientific, Waltham, MA, USA) supplemented with 1% Glutamine (Gibco, Thermo Fisher Scientific, Waltham, MA, USA), 1% Penicillin/Streptomycin (Gibco, Thermo Fisher Scientific, Waltham, MA, USA), and 10% FBS (Gibco, Thermo Fisher Scientific, Waltham, MA, USA). Neuroblastoma cell lines (iLIN28B and CTRL) were kept at 37 °C, 5% CO_2,_ and 95% humidity, and 1 µM doxycycline (DOX) (Sigma‐Aldrich, St. Louis, MO, USA) was administered for 7 d.

### Gene Expression Analysis

Total RNA was extracted using Trizol Reagent (Invitrogen, Thermo Fisher Scientific, Waltham, MA, USA) and Zymo Direct‐zol (Zymo Research, Freiburg im Breisgau, Germany), as described elsewhere.^[^ ^6^ ^]^ The quantification was performed using Qubit (Invitrogen, Thermo Fisher Scientific, Waltham, MA, USA). Quality and integrity of RNA were assessed using the RNA Nano Assay on the Agilent 2100 Bioanalyzer (Agilent Technologies, Tokyo, Japan). One hundred ng of total RNA was used for the transcription, hybridization, and biotin labeling using the GeneChip Whole Transcript PLUS Reagent Kit Manual Target Preparation for GeneChip Whole Transcript Expression Arrays protocol (Affymetrix, Thermo Fisher Scientific, Waltham, MA, USA). The manufacturer's instructions were followed for all protocols. Samples were hybridized using the Human Clariom S Gene Chip Cartridge Array.

The.CEL files were normalized using the robust multiarray averaging expression measure of Affy‐R package by Transcriptome Analysis Console (TAC Software v.4.0.2.15, Thermofisher). To identify differently expressed genes (DEG), significance analysis of micorarray^[^ ^47^ ^]^ (SAM, samr package) in R‐Bioconductor was applied between iLIN28B and CTRL cells. Estimated percentage of false‐positive predictions (i.e., false discovery rate, FDR) was obtained with 1000 permutations and genes with an FDR < 0.05 were considered significant. DEG were used to perform overrepresentation analysis (Biological Process and Cellular Component from Gene Ontology database) using ClusterProfiler package (v.4.4.4)^[^ ^48^ ^]^ and enriched terms were selected with p.adjusted <0.1 and Benjamini‐Hochbelg (BH) correction. To reduce GO:BP terms complexity, treeplot() function was used and pairwise similarities of the enriched terms was calculated by pairwise_termsim() function with default Jaccard's similarity index. A complete method was applied in the treeplot function to generate hierarchical clustering analysis. Moreover, rrvgo package^[^ ^49^ ^]^ was used to reduce lists of GO:CC terms by grouping them based on semantic similarity with a threshold = 0.7. Scatter plot visualized the results as GO terms as scattered points and distance between points as the similarity between terms and x‐axis and y‐axis as the first 2 components of applying a PCoA to the dissimilarity matrix. The size of points represents the number of genes for each term. Gene Set Enrichment Analysis (GSEA)^[^ ^50^ ^]^ was performed using GSEAv4.0 with probe sets ranked by signal‐to‐noise ratio and statistical significance determined by 1000 permutations. Gene sets permutations (< 7 replicates in each class) were used to enable direct comparisons between iLIN28B and CTRL cells. MgSigDataBase derived from Hallmark curated dataset were selected to obtain the enrichment gene sets. Cor() function in Bioconductor was used to identify LIN28B correlated genes in obtained transcriptome data. The data supporting the findings of this study are available in GEO, reference number GSE252806.

### Fireplex microRNA Assay

A *let‐7* miRNAs profile of 1.0 × 10^5^ iLIN28B (*n* = 3) and CTRL cells (*n* = 3) was obtained through a FirePlex miRNA analysis service in outsource (Abcam) as part of a panel specific for the human neuronal tissues (cat. N. ab218371). Data was analyzed with the FirePlex Analysis Workbench software, available on the Abcam website at www.abcam.com/FireflyAnalysisSoftware, and R. Normalized data were log10 transformed, scaled and centered using “scale” function from the “base” R package. *let‐7* family miRNAs were analyzed using “heatmap3” function from the ′heatmap3’R package.^[^ ^51^ ^]^

### Quantitative Real‐Time PCR (qPCR)

One microgram of total RNA was used for cDNA synthesis using the Superscript III First‐Strand Synthesis System (Invitrogen, Thermo Fisher Scientific, Waltham, MA, USA) according to the manufacturer's instructions. The expression of specific transcripts was analyzed by qPCR using the SYBR Green PCR Master mix (Applied Biosystems, Thermo Fisher Scientific, Waltham, MA, USA) in the 7900HT Fast Real‐Time PCR System with 96‐well block module. Samples were run in triplicate and the expression of GAPDH was used as an internal control gene for normalization. Data were quantified using the ∆∆Ct method. Primer sequences were designed using the Primer 3 software and are listed in Table S1 (Supporting Information).

### Extracellular Matrix Coatings

Fibronectin (FN) (Corning, New York, NY, USA; diluted to 10 µg mL^−1^ in PBS) and Laminin (LM) (Sigma‐Aldrich, St. Louis, MO, USA; diluted to 10 µg mL^−1^ in Milli‐Q water) were applied to the plate surface for 3 h at 37 °C. Vitronectin (VTN) (Sigma‐Aldrich, St. Louis, MO, USA; diluted to 10 µg mL^−1^ in deionized water supplemented with 0.1% BSA) was applied to the plate surface for 2 h at 37 °C at first and then transferred at 4 °C overnight. The coatings were washed with PBS before use. Collagen I (COL I) was used as commercially available pre‐coated plates (Gibco, Thermo Fisher Scientific, Waltham, MA, USA). Cell adhesion capacity was measured after 24 h of cell growth on different coatings; non‐adherent and loosely attached cells were removed by gently washing the wells with PBS. Five randomly selected fields were taken for each condition to quantify the mean number of attached cells.

### MMP‐2/9 Activity Assay

The amount of active MMP‐2, and MMP‐9 was determined by the fluorogenic Innozyme gelatinase activity assay kit (Sigma‐Aldrich, St. Louis, MO, USA) as recommended by the manufacturer. Briefly, culture supernatants collected after 72 h on coatings, were incubated with substrate working solution for 3 h at 37 °C. Fluorescence was measured at an excitation wavelength of 320 nm and an emission wavelength of 405 nm in a microplate reader (Tecan, Mannedorf, Switzerland), and the concentration (ng mL^−1^) of active MMP‐2, and MMP‐9 was calculated indirectly from a standard curve for each assay.

### Immunoblot Assay

Total proteins were extracted with a Lysis Buffer (Biosource International, Thermo Scientific, Waltham, MA, USA) and protein concentration was determined using the bicinchoninic assay (BCA) method (Pierce BCA Protein Assay Kit, Thermo Scientific, Waltham, MA, USA). Proteins from CM were collected and centrifuged at 2000 rpm for 10 min at 4 °C. The samples were then concentrated using the Amicon Ultra 0.5 mL Centrifugal Filters (Merck Millipore, Burlington, MA, USA) and stored immediately at ‐80 °C. Cytosol and nuclear protein fractions were obtained as described elsewhere.^[^ ^52^ ^]^ A total of 20 µg of proteins was loaded for each sample on Criterion TGX Stain‐Free Precast 4–20% gradient polyacrylamide gels (Bio‐Rad, Hercules, CA, USA), transferred to activated nitrocellulose membrane (PVDF Membrane, TransBlot‐ Turbo, Bio‐Rad through the Trans‐Blot Turbo Transfer semidry System (Bio‐Rad, Hercules, CA, USA). Membranes were blocked using 3% BSA (Sigma‐Aldrich, St. Louis, MO, USA) for 1 h and probed with the primary and secondary antibodies listed in Table S2 (Supporting Information). The binding of the antibodies with the specific proteins was detected using the substrate ECL Select Western Blotting Detection Reagent (GE Healthcare, Chicago, IL, USA) and iBright FL1500 Imaging System (Invitrogen, Thermo Fisher Scientific, Waltham, MA, USA) for the chemiluminescence detection.

### Immunofluorescence

CTRL and iLIN28B cells were fixed using 4% paraformaldehyde (PFA; Sigma‐Aldrich, St. Louis, MO, USA) for 10 min and permeabilized with 0.1% Triton X‐100 for 10 min. The coverslips were blocked in 5% BSA for 1 h at room temperature. The primary and secondary antibodies used in this study are listed in Table S2 (Supporting Information). Nuclei were counterstained with DAPI (1:1000; Invitrogen, Thermo Fisher Scientific, Waltham, MA, USA). Phalloidin‐TRITC (Sigma‐Aldrich, St. Louis, MO, USA) was diluted at 1:400 in 3% BSA and used to detect F‐Actin. Each coverslip was subsequently mounted on glass slides using 80% glycerol and analyzed using a confocal microscope (Zeiss LSM 800, Oberkochen, Germany). Images were analyzed using ImageJ software (National Institutes of Health, Bethesda, MD, USA).

### EdU Proliferation Assay

After 72 h on coatings, CTRL and iLIN28B cells were incubated with 10 µm EdU substrate solution (BaseClick GmbH, Germany) for 4 h at 37 °C, followed by fixation in 4% PFA (Sigma‐Aldrich, St. Louis, MO, USA) for 15 min and permeabilization with 0.5% Triton X‐100 for 20 min. EdU488 incorporation was detected via a fluorescent azide reaction using the kit's click chemistry reagents. Then, nuclei were counterstained with DAPI (1:1000; Invitrogen, Thermo Fisher Scientific, Waltham, MA, USA). For each experimental condition, five random fields were acquired with a confocal microscope (Zeiss LSM 800, Oberkochen, Germany) and the images were analyzed using AIVIA software (Leica). The proliferation rate was quantified as the proportion of proliferating (green) cells to total (blue) cells, expressed as a percentage.

### Retinoic Acid Treatment

13‐RA (MCE MedChemExpress, Monmouth Junction, NJ, USA) was dissolved in DMSO and diluted in cell culture medium to obtain final concentrations of 10 and 50 µm. The final concentration of DMSO in all experiments never exceeded 0.1%. Cells were treated for 72 h and the RNA was collected for qPCR analysis as described above.

### Preparation of Tumor Cell Conditioned Medium (CM)

Cell culture supernatants were collected from CTRL and iLIN28B cells by replacing the complete RPMI medium with RPMI supplemented with 5% BIT for 48 h. The CM was centrifuged at 1800 rpm for 10 min at 4 °C. For neutralization experiments, CM was preincubated with the antibody against human IGF2 (0.8 µg mL^−1^; AF‐292‐NA; R&D Systems, Minneapolis, MN, USA) overnight at 4 °C.

### In Vitro Endothelial Cell Tube Formation Assay

Two hours before the treatment with CM, 2 × 10^3^ endothelial cells resuspended in 100 µL of complete Medium 200 were seeded in a 96‐well plate precoated with Matrigel (R&D Systems, Minneapolis, MN, USA). Then, Medium 200 was replaced with 100 µL of CM derived from either CTRL or iLIN28B cells. The tube formation ability of endothelial cells was examined by phase‐contrast microscopy after 24 h. The mean number of meshes and nodes of the tubular structures was quantified using ImageJ software (National Institutes of Health, Bethesda, MD, USA). Endothelial cells were treated with 0.1 µm of IGF1R inhibitor PPP (MCE MedChemExpress, Monmouth Junction, NJ, USA) to impede IGF2 action and effect on cell behavior was monitored 24 h post‐treatment. When indicated, the PPP was used in combination with the IGF2‐neutralizing antibody to study their co‐effect. Control cells were treated with DMSO used as a drug vehicle.

### Scratch Assay and Time‐Lapse Migration

Endothelial cells (2.5 × 10^4^) were seeded into each well of the two‐cell culture insert separated by a 500 µm thick wall (IBIDI, Grafelfing, Germany). Cells were incubated at 37 °C overnight, then the insert was removed, and cells were washed with PBS to remove non‐attached cells. Each well was filled with CM derived from either CTRL or iLIN28B cells. Images were recorded at 0 and 24 h using an inverted optical microscope equipped with a camera (Nikon, Tokyo, Japan). The wound closure was analyzed with Fiji software (National Institutes of Health, Bethesda, MD, USA). For time‐lapse migration upon coatings, CTRL and iLIN28B neuroblastoma cells were analyzed with an Axio Observer microscope (Zeiss, Oberkochen, Germany) equipped with an incubator maintained at 37 °C with 5% CO_2_. Cell motility was recorded at 10 min intervals over a 12 h duration, and their trajectories documented using the “manual tracking” plug‐in in ImageJ software (National Institutes of Health, Bethesda, MD, USA).

### Transendothelial Migration Assay

1 × 10^5^ endothelial cells were pre‐stained with Hoechst (Sigma Aldrich, St. Louis, MO, USA) and seeded onto the upper chamber of a 3 µm transwell (Corning, New York, NY, USA) pre‐coated with 0.1% gelatin to form an endothelial monolayer. Endothelial cells were activated with 10 ng mL^−1^ TNFα (Cell Biolabs, San Diego, CA, USA) for 6 h at 37 °C. The day after, 5 × 10^4^ CTRL and iLIN28B cells pre‐labeled with calcein were seeded in the same chamber at the top of the endothelial cells’ monolayer. An FBS (Gibco, Thermo Fisher Scientific, Waltham, MA, USA) gradient was generated by placing the Medium 200 supplemented with 1% FBS in the upper chamber and the Medium 200 supplemented with 20% FBS in the lower chamber. The transmigration ability of CTRL and iLIN28B cells was recorded with a live‐imaging microscope (Zeiss, Oberkochen, Germany). The analysis was conducted at 24, 48, and 72 h by taking Z‐stack images. Quantification of migrated cells was performed using ImageJ software (National Institutes of Health, Bethesda, MD, USA) by analyzing five randomly selected fields per transwell.

### Generation of Cell Spheroids

For generation of neuroblastoma spheroids, a suspension of either CTRL or iLIN28B cells was loaded into an ultra‐low attachment 96‐wells round‐bottomed plates (Corning, New York, NY, USA) at a final density of 2.5 × 10^4^ cells per well. Cell aggregation was facilitated by centrifugation of the plate at 1000 rpm for 5 min. After 48 h of culture, the FBS concentration was reduced to 2%. Once the spheroids were completely formed upon 7 d of culture they were used for experiments. Three independent groups of spheroids were used. Growth kinetics of neuroblastoma spheroids were evaluated by taking pictures at 24, 48, 72 h, and 7 d of culture using a conventional inverted microscope (Nikon, Tokyo, Japan) The volume of each spheroid was determined with ImageJ software (National Institutes of Health, Bethesda, MD, USA) and represented in mm^3^. For the generation of multi‐cellular spheroids (endothelial and neuroblastoma co‐culture), CTRL and iLIN28B cells were labeled with Vybrant DiL Cell‐Labeling Solution (Invitrogen, Thermo Fisher Scientific, Waltham, MA, USA) and seeded to form spheroids as previously described. After 3 d, each spheroid was transferred into a 96‐well black plate (Thermo Fisher Scientific, Waltham, MA, USA) and resuspended in 50 µL of VitroGel hydrogel (TheWell Bioscience, North Brunswick Township, NJ, USA) containing 5 × 10^4^ endothelial cells pre‐labeled with Vybrant DiO Cell‐Labeling Solution (Thermo Fisher Scientific, Waltham, MA, USA). After 15 min at room temperature, the gel‐like cell suspension was covered with 50 µL of fresh Medium 200. The obtained 3D co‐cultures were daily monitored for the formation of tube‐like structures around the spheres. Ten spheroids for each experimental condition were analyzed. Images were taken after 7 d of co‐culture through confocal microscopy (Zeiss LSM800, Oberkochen, Germany).

### Migration and Invasion Assays with Spheroids

Neuroblastoma spheroids generated through low adhesion supports were embedded into a solution of 50% Matrigel (R&D Systems, Minneapolis, MN, USA) / 50% complete RPMI and then incubated for 3 d. The cell invasion was monitored at 0, 24, 48, and 72 h through light microscopy, and the invaded area was measured as a function of time using ImageJ software (National Institutes of Health, Bethesda, MD, USA). To assess migration in a 3D setting, CTRL and iLIN28B spheroids were transferred in pre‐coated plates and allowed to adhere to the indicated ECM proteins. Images were taken at 0, 24, 48, and 72 h using a light microscope. The distance migrated was calculated with ImageJ software (National Institutes of Health, Bethesda, MD, USA).

### Cell Viability

The cell viability was assessed with a Live/Dead Assay (Invitrogen, Thermo Fisher Scientific, Waltham, MA, USA) following the manufacturer's instructions. Briefly, live cells were identified using 0.1 µm calcein‐AM, while dead cells were identified using 4 µM ethidium bromide. Samples were imaged through a LSM800 confocal microscope (Zeiss, Oberkochen, Germany). A Resazurin assay (Sigma‐Aldrich, St. Louis, MO, USA) was used to determine the effects of PPP treatment on endothelial cells. Resazurin was added to the samples and incubated for 3 h at 37 °C. Absorbance was measured at 570 nm using a microplate reader (Tecan, Mannedorf, Switzerland). The percentage of cell viability was normalized to the values obtained for the control cells (treated with DMSO vehicle).

### Nascent RNA Analysis

Nascent RNA was assessed using the Click‐iT Nascent RNA Capture kit (Thermo Fisher Scientific, Waltham, MA, USA). Briefly, the cells were incubated with 0.2 mm 5‐ethynyl uridine (EU) for 4 h. Cells were harvested and total RNA was extracted using Trizol reagent as described earlier. EU‐labeled RNA was then biotinylated and captured using the Dynabead MyOne Streptavidin T1 magnetic beads, and retrotranscribed using the Superscript II Reverse Transcriptase (Invitrogen, Thermo Fisher Scientific, Waltham, MA, USA) according to the manufacturer's instructions. The expression of specific transcripts was analyzed by qPCR using the SYBR Green PCR Master mix (Applied Biosystems, Thermo Fisher Scientific, Waltham, MA, USA) using *GAPDH* as an internal control gene (Table S1, Supporting Information).

### Human‐IGF Signaling Array

The human IGF Signaling Array 1 (Quantibody; RayBiotech, Peachtree Corners, GA, USA) was performed using 100 µL of CM from CTRL and iLIN28B cells following the manufacturer's instructions. The experiment was run in biological duplicate. Imaging was performed using the Quantibody Array Testing Software (RayBiotech, Peachtree Corners, GA, USA).

### RNA Immunoprecipitation Assay (RIP)

The protocol for RNA immunoprecipitation has been adapted from.^[^ ^53^ ^]^ In brief, a pellet of 3×10^6 iLIN28B cells was resuspended in 20 µL of Polysome Lysis Buffer and immediately frozen in liquid nitrogen. A total amount of 100 µg protein extracts was incubated overnight at 4 °C with the LIN28B antibody or the Rabbit IgG negative control (Table S2, Supporting Information) on gentle rotation. The remaining amount of iLIN28B‐derived protein‐RNA extracts was lysed in 900 µL Trizol and frozen at ‐80 °C for input normalization. The next day, the samples incubated with antibodies were mixed with 1.5 mg Dynabeads Protein G beads (Invitrogen, Thermo Fisher Scientific, Waltham, MA, USA) and left for 30 min on gentle rotation. Beads were washed four times with 1 mL of ice‐cold NT2 buffer and resuspended in 100 µL of NT2 buffer supplemented with 30 µg of proteinase K (Roche, Basel, Switzerland) to release the ribonucleoprotein components. Samples were incubated for 30 min at 55 °C. Next, trizol was added directly to the beads to isolate RNA from complexes. Isolated RNA was retrotranscribed using the high‐capacity cDNA synthesis kit (Thermo Fisher Scientific, Waltham, MA, USA). The expression of specific transcripts was analyzed by qPCR using the SYBR Green PCR Master mix (Applied Biosystems, Thermo Fisher Scientific, Waltham, MA, USA). The expression of *GAPDH* was used as internal control gene (Table S1, Supporting Information) and data were analyzed using the comparative ΔΔCt method. The abundance of the indicated mRNAs in iLIN28B immunoprecipitated (IP) and IgG IP samples was calculated by normalizing each mRNA level to its levels detected in the input samples.

### Zebrafish Xenograft Models

The transgenic line Tg(KDRL:GFP) was staged and maintained as previously described,^[^ ^54^ ^]^ according to the local and Italian ethical guidelines. Zebrafish embryos at 48 h post fertilization (hpf) were anesthetized with 0.003% Tricaine (Sigma‐Aldrich, St. Louis, MO, USA) diluted in fish water. The CTRL and iLIN28B cells were pre‐labeled with the Vybrant DiL Cell‐Labeling Solution (Invitrogen, Thermo Fisher Scientific, Waltham, MA, USA) according to the manufacturer's instructions. In the first xenotransplantation model, ten nanoliters of Matrigel (R&D Systems, Minneapolis, MN, USA) containing 100–150 cells were implanted into each zebrafish embryo in the perivitelline space by using an electronically regulated air‐pressure microinjector (WPI, Sarasota, FL, USA).^[^ ^25^ ^]^ In the second xenotransplantation model, the same amount of tumor cells was resuspended in PBS and injected into the neural tube of 72 hpf‐embryos as described by Kocere and co‐workers.^[^ ^55^ ^]^ Subsequently, the embryos were inspected for the presence of fluorescent tumor cells through a fluorescent stereomicroscope (Nikon, Tokyo, Japan) to assure sample‐to‐sample uniformity and to exclude the embryos with <50 cells. Each embryo was transferred to a 96‐well plate containing 200 µL of fish medium and incubated at 34 °C for 24 h. For in vivo imaging, anesthetized zebrafish embryos were analyzed using a Zeiss LSM 800 confocal microscope (Zeiss, Oberkochen, Germany). Images were processed using ImageJ software (National Institutes of Health, Bethesda, MD, USA) for merging Z‐stacks.

### Alkaline Phosphatase Assay

The whole‐mount staining for endogenous alkaline phosphatase (AP) activity was performed as previously described.^[^ ^25^ ^]^ Briefly, embryos were fixed with 4% PFA in PBS overnight at 4 °C. Embryos were then dehydrated in 25%, 50%, 75%, and 100% methanol (in PBS, 5 min each step) and rehydrated stepwise to PBS/0.1% Tween 20. Embryos were equilibrated in developing buffer (0.1 m Tris‐HCl pH = 9.5, 50 mm MgCl_2_, 0.1 m NaCl, and 0.1% Tween 20) for 30 min at room temperature and incubated with the solution of 18.75 mg mL^−1^ nitro blue tetrazolium chloride and 9.4 mg mL^−1^ 5‐bromo‐4‐chloro‐3‐indolyl‐phosphate, toluidine‐salt in 67% DMSO (v/v; Sigma‐Aldrich, St. Louis, MO, USA) to obtain vessels labeling. Embryos were photographed under a stereomicroscope equipped with a digital camera (Nikon, Tokyo, Japan) to analyze vessel sprouting.

### Statistical Analysis

The experiments were run in three biological replicates except for the human‐IGF signaling array which was carried out in duplicate. Data are presented as the mean number ± standard error (S.E.M.). Statistical analyses were performed using the Student's *t*‐test and graphs were created using GraphPad Prism 8 (GraphPad, La Jolla, CA). Statistical significance was indicated as * for *p* < 0.05 and ** for *p* < 0.01.

### Ethics Approval and Consent to Participate

Fish handling procedures and care maintenance were accomplished in accordance with the EU Directive 2010/63/EU on the protection of animals used for scientific purposes. All experiments were conducted in non‐feeding zebrafish embryos and were approved by the local OPBA (Organismo per il Benessere e Protezione Animale) of the Istituto di Ricerca Pediatrica Città della Speranza.

## Conflict of Interest

The authors declare no conflict of interest.

## Author Contributions

Conception and design: D.C. and S.A.; development of methodology: D.C. and S.A.; analysis and interpretation of data: D.C., S.M., S.B., C.Z., A.D., and S.A.; writing: D.C. and S.A.; revision of the manuscript: A.B. and M.M. All authors have read and agreed to the published version of the manuscript.

## Supporting information



Supporting Information

## Data Availability

The datasets analyzed during the current study are available in GEO, reference number GSE252806.^[^ ^45^ ^]^
