# Supplementary material for: LIN28B Promotes Cancer Cell Dissemination and Angiogenesis
Source: Adv Biol (Weinh). 2025 Jul 18;9(11):e00730. doi: 10.1002/adbi.202400730 (PMC12624821; doi:10.1002/adbi.202400730)
Supplement: Supplementary file 1 — Supporting Information [file ADBI-9-e00730-s001.docx]

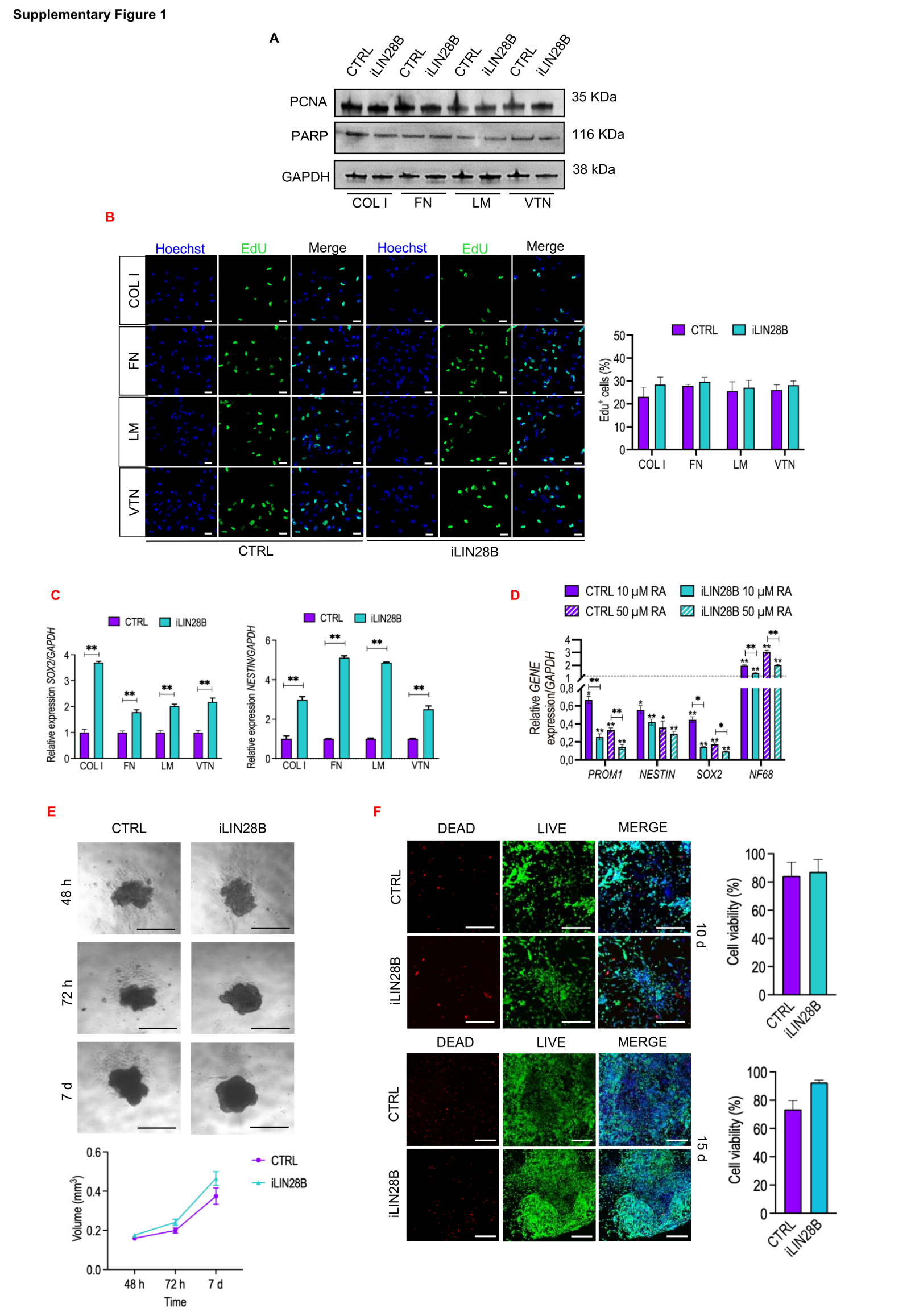


**Supplementary Figure S1**. (A) Western blot analysis for the indicated markers on CTRL and iLIN28B cells upon the growth on the indicated coating proteins. An antibody against GAPDH was used as a loading control. The molecular weights are indicated in Kilodaltons (KDa). (B) In vitro cell proliferation activity of CTRL and iLIN28B cells after three days upon the indicated coatings. The proliferating cells were determined with EdU (green) whereas all the nuclei were labeled with Hoechst (blue). Scale bar, 100 μm. Right: quantification of EdU^+^ cells (%). (C) Real-time qPCR analysis for *SOX2* and *NESTIN* mRNAs in CTRL and iLIN28B cells. The expression of *GAPDH* was used for data normalization. **P<0.01 compared to CTRL_S_ (Student’s t-test). (D) Real-time qPCR analysis for the indicated stem cell and differentiation mRNAs in CTRL and iLIN28B cells. GAPDH expression was used for data normalization. Statistical comparisons were conducted relative to control (CTRL) cells treated with DMSO (represented by the dotted line, set at a relative expression level of 1), as well as among the specified experimental groups. *P<0.05; **P<0.01 compared to CTRL_S_ (Student’s t-test). (E) Top: representative images of CTRL and iLIN28B spheroids grown in the ultra-low attachment 96-well round-bottomed plates. Pictures were taken at 48, 72 hours (h) and 7 days (d) of culture to monitor spheroid growth kinetic. Bottom: quantification of the spheroid volumes over time (mm^3^). Scale bar, 1 mm. (F) Live and dead cell viability of CTRL and iLIN28B spheroids grown for 10 (upper panel) and 15 (lower panel) days (d), respectively. Right: quantification of cell viability. Scale bar, 200 μm. Data are presented as the mean number ± standard error (S.E.M.). *P<0.05; **P<0.01 compared to CTRL_S_ (Student’s t-test).


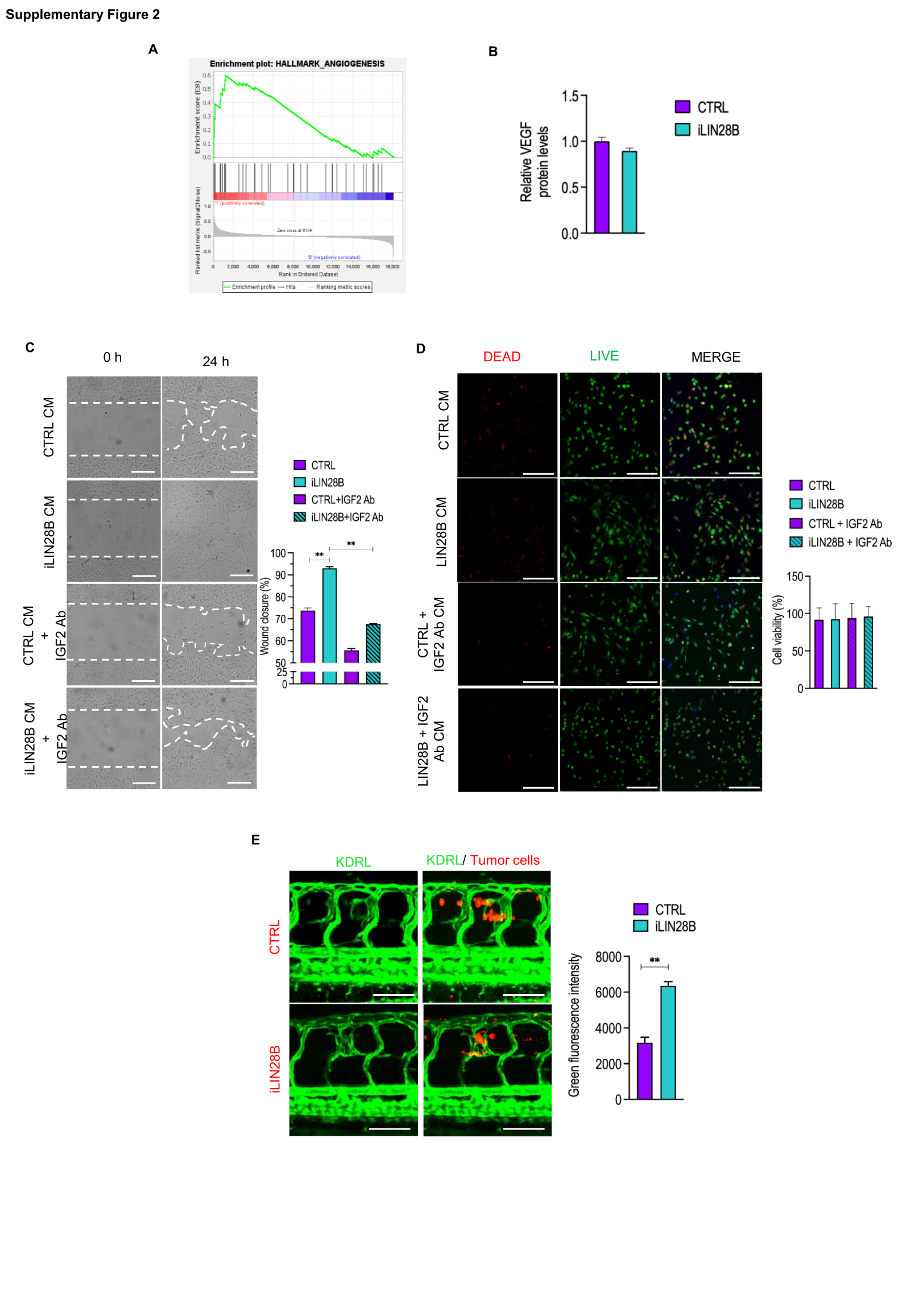


**Supplementary Figure S2.** Gene set enrichment analysis (GSEA) of iLIN28B-dependent mRNAs for gene sets belonging to the process of angiogenesis. Each black line represents a single gene in the gene set. Significance set at FDR < 0.05. FDR - false discovery rate. (B) Quantification of relative human VEGF protein levels in iLIN28B cells, normalized to the CTRL counterpart. (C) Left: representative brightfield images of a scratch assay with endothelial cells treated for 24 hours (h) with CTRL and iLIN28B conditioned media (CM) treated with IGF2 blocking antibody (IGF2 Ab) and picropodophyllin (PPP) when indicated. Scale bars, 200 μm. The leading fronts are highlighted with white dashed lines. Right: quantification of the percentage (%) of wound closure. (D) Left: live (green) and dead (red) staining of endothelial cells treated for 24 hours with CTRL and iLIN28B conditioned media (CM) pre-incubated with the IGF2 blocking antibody (IGF2 Ab) when indicated. Nuclei were counterstained with DAPI (blue). Scale bar, 200 µm. Right: quantification cell viability (%) in response to the indicated treatments. (E) Left: confocal microscopy images of tumor vasculature of the neural tube stimulated by the xenotransplantation of CTRL and iLIN28B cells (red). Scale bar, 100 μm. Right: quantiﬁcation of green fluorescence intensity deriving from blood vessels. *n=*30. Data are presented as the mean number ± standard error (S.E.M.). *P<0.05; **P<0.01 compared to CTRLs (Student’s t-test).

| **Gene name** | **Primer sequences** |
| --- | --- |
| PROM1 | (F) ACCTTGAAGAGCTTGCACCA |
|  | (R) ATGGATGCACCAAGCACAGA |
| SOX2 | (F) GCCGAGTGGAAACTTTTGTCG |
|  | (R) GGCAGCGTGTACTTATCCTTCT |
| NESTIN | (F) CTTCCCTCAGCTTTCAGGAC |
|  | (R) TCAGGACTGGGAGCAAAGAT |
| NF68 | (F) CAAGGACGAGGTGTCCGAG |
|  | (R) CCCGGCATGCTTCGA |
| GAPDH | (F) GTGGAGTCTACTGGTGTCTTC |
|  | (R) GTGCAGGAGGCATTGCTTACA |
| COL6A2 | (F) CCTCTGCCCGGACCCTCA |
|  | (R) CACGGACCCCGAGAAAACCT |
| IGF2 | (F) GTGCTACCCCCGCCAAGT |
|  | (R) TGGACTGCTTCCAGGTGTCA |
| IGF2 - P1 | (F) TGCTTTGGTGGTGACTGCTAA |
|  | (R) GAAACTGCCTGGACGATGATC |
| IGF2 - P3 | (F) ATTACACGCTTTCTGTTTCTCTCC |
|  | (R) AAATGAGGTCAGCTGTTGTATCAAG |
| IGF2 - P4 | (F) TCTCCTGTGAAAGAGACTTCCAG |
|  | (R) CAAGAAGGTGAGAAGCACCAG |
| ACSL6 | (F) CGCTACATCATCAATACAGCGG |
|  | (R) GCATGGACTTAATGACCACCC |
| SREPB-1A | (F) GGAGGGGTAGGGCCAACGGCCT |
|  | (R) CATGTCTTCGAAAGTGCAATCC |
| LIN28B | (F) GGATTTGGATTCATCTCCAATGA |
|  | (R) GAATTCCACTGGTTCTCCTTCT |

**Supplementary Table S1.** List of primers used in qPCR analyses.


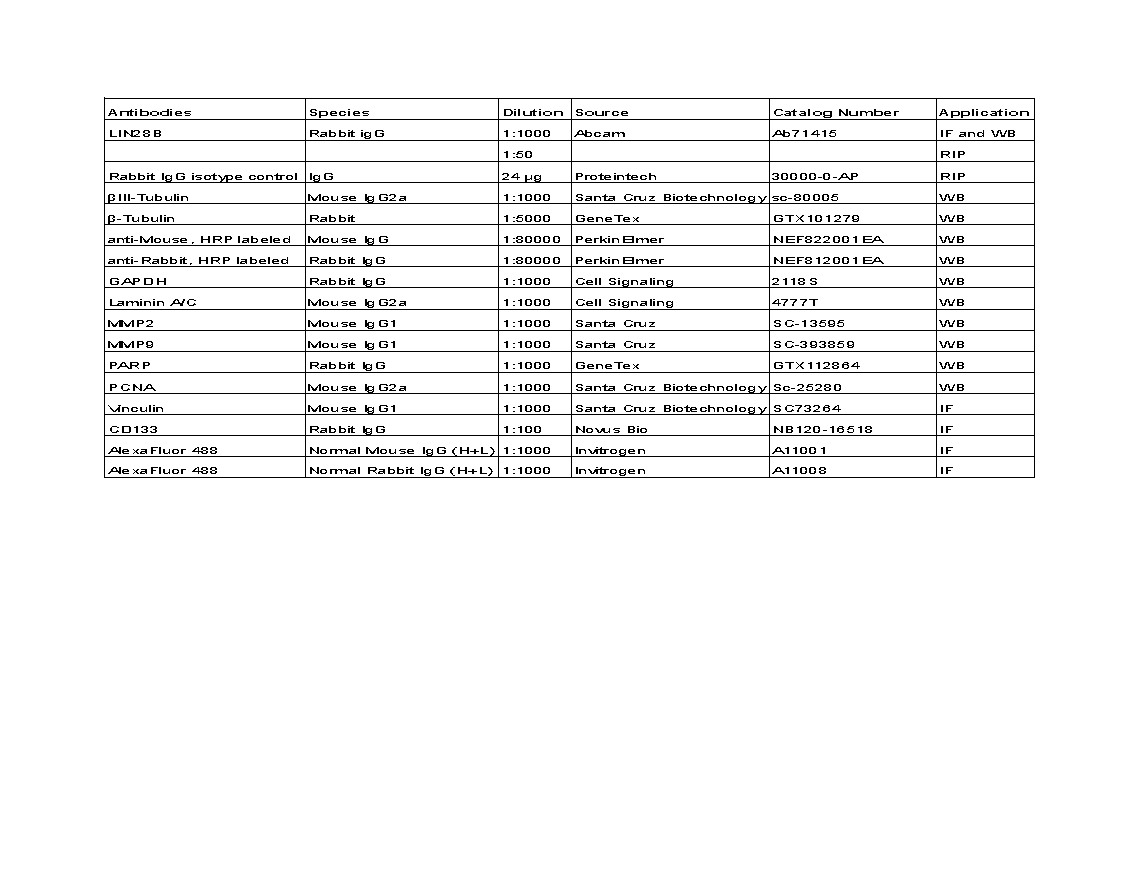


**Supplementary Table S2.** List of primary and secondary antibodies used for immunofluorescence (IF), immunoblot (IB), and RNA immunoprecipitation (RIP) analysis.
